# 

**Published:** 1861-10

**Authors:** 


### CONTENTS.

| ART. | | PAGE |
|---|---|---|
| | Quarterly Retrospect | xlvii—lvi |
| I. | The Study of Medicine | 537 |
| II. | The Case of the Egyptian Frigate at Liverpool, with Remarks on the Causation of Fevers, &c. By Gavin Milroy, M.D., F.R.C.P. | 552 |
| III. | The Æsthetics of Suicide | 563 |
| IV. | Female Physicians | 586 |
| V. | Orientalism | 603 |
| VI. | Swedenborg's Dreams | 612 |
| VII. | On the Educational Treatment of Cretinism. By J. Mundy, M.D., of Moravia | 626 |
| VIII. | Medical Students:—A New Generation | 636 |
| IX. | The State of Lunacy in England | 642 |
| X. | Unrecognised Insanity | 659 |
| XI. | On Hallucinations in their Relation to Medical Jurisprudence. By A. Brierre de Boismont | 668 |
| XII. | Medical Gossip | 682 |
| XIII. | Literary Gossip and Record | 692 |
| | Foreign Medico-Psychological Literature | 712 |
| | Obituary | 727 |
| | Index | 729 |

No. V. will be published on the 1st of January.

